# Drug Allergy Profile From a National Drug Allergy Registry

**DOI:** 10.3389/fphar.2020.555666

**Published:** 2021-01-08

**Authors:** Mona Al-Ahmad, Jusufovic Edin, Fardous Musa, Tito Rodriguez-Bouza

**Affiliations:** ^1^ Al Rashed Allergy Centre, Ministry of Health, Kuwait; ^2^ Microbiology Department, Faculty of Medicine, Kuwait University, Kuwait; ^3^ Medical Faculty, University of Tuzla, Tuzla, Bosnia and Herzegovina

**Keywords:** drug allergy, registry, hypersensitivity, allergy, NSAIDs, penicillin

## Abstract

**Background:** Drug hypersensitivity reactions (DHRs) are among the most frequent reasons for consultation in allergy departments and are becoming more common due to increasing prevalence and case complexity.

**Objective:** To describe the most common drugs associated with clinical reactions, diagnostic methods used, and outcomes of allergic evaluations of a national drug allergy registry over a 12-year period were used.

**Methods:** An observational, prospective, patient’s data registry-based study was conducted to analyze all referrals to the drug allergy outpatient clinics at Al-Rashed Allergy Center, Kuwait, between 2007 and 2019. Demographics, description of DHRs, and results of allergy tests to potential causative medications were reviewed. Diagnostic methods were focused mainly on skin tests (STs) and drug provocation test (DPT), when indicated.

**Results:** We evaluated 1,553 patients with reported DHRs. The mean age of the population was 41.52 ± 16.93 years, and the study population consisted of 63.7% female patients. Hypersensitivity was finally confirmed in 645 (41.5%) of patients, probable in 199 (12.8%), and not confirmed/nonallergic in 709 (45.6%) patients. Anti-inflammatory drugs and analgesics contributed to 39.22% of all confirmed drug allergies, followed by antibiotics 38.1% (β-lactam antibiotics (BLs) constituted 73.98% of all antibiotics and 28.21% of all drugs), anesthetics 1.8%, and radio-contrast media 0.31%. The majority of reactions were non-immediate 51.44%. The most commonly presenting symptoms among confirmed patients were urticaria 57.80%, angioedema 42.50%, respiratory symptoms 47.60%, and erythema 33.60%. Symptoms of anaphylaxis/anaphylactic shock were reported by 284 patients (44.00%) among confirmed cases. The most common method of diagnosis was a positive clinical history (54.4% in BLs and 90.45% in nonsteroidal anti-inflammatory drugs (NSAIDs). Among confirmed allergy to BLs, a positive ST was obtained in 31.9% of patients and positive DPT in 13.7%.

**Conclusion:** NSAIDs and antibiotics, mainly BLs, are the most commonly implicated in confirmed allergy. In both confirmed and not confirmed/nonallergic cases, BLs are the most frequently involved DHRs which are mainly immediate, and the most commonly presenting symptoms were urticaria, angioedema, and respiratory symptoms. Diagnosis was confirmed mainly by a positive clinical history and when indicated, by positive STs or a DPT.

## Introduction

A drug hypersensitivity reaction (DHR) can be defined as an adverse drug reaction (ADR), with an immunological etiology, to an otherwise safe and effective therapeutic agent (Park and Demoly, 2012; Böhm and Cascorbi, 2016). Type I hypersensitivity (IgE-mediated) reactions are the most studied among other DHRs that were described by Gell and Coombs (Demoly et al., 2008). DHRs are of significant concern for clinicians and patients as suspected cases may result in avoidance of first-line medications like in cases of suspected *β*-lactam antibiotic (BL) allergy that leads to worse outcomes and increased cost (Macy and Contreras, 2014; Su et al., 2017; Sousa-Pinto et al., 2018), and in consequence, both under- and overdiagnosis of DHRs are potential challenges in everyday practice. Although *in vivo* and *in vitro* testing including the gold standard drug provocation test (DPT) can confirm the diagnosis, and clinicians have to challenge problems such as the lack of standardized tests to most of the medications, the contraindication for DPT in severe cases, or patient refusal to undergo a DPT with the culprit drug. These problems push clinicians to accept the diagnosis of drug allergy based on clinical history alone on the cases that there is no standardized test or DPT is not as suitable option. Throughout the years, two main groups of drugs have consistently remained prevalent worldwide, BLs and nonsteroidal anti-inflammatory drugs (NSAIDs), with different clinical presentations like cutaneous symptoms of urticaria, angioedema, and respiratory symptoms, among others (Demoly et al., 2014). Many factors affect the DHRs, some are related to the drug itself as the ability to act as a hapten, prohapten, or binding to immune receptors and others to patient factors like female sex, age, history of drug reactions, concomitant infections, or genetics (HLA genotypes) (Gamboa, 2009; Thong and Tan, 2011).

Despite the fact that many studies utilize data from patient’s database and electronic medical records, there are not many publications on specifically drug allergy databases, and none of the previously published ones belong to the Middle East region. The reasons might be related to the difficulties and challenges of maintaining and following up patients in a registry-based format. This might be due to the need of a specific database on drug allergy using common standardized procedures (Bousquet et al., 2009). The most remarkable existing database from Europe is the Drug Allergy and Hypersensitivity Database (DAHD) that has provided information regarding cross-reactivity with cephalosporins in confirmed allergic patients to BL (Sidoroff et al., 2010), and of other BLs in proven allergy to cefazolin (Pipet et al., 2011), the need for DPT after negative skin testing (Bousquet et al., 2008), risk of systemic reactions during skin testing (Co Minh et al., 2006), the accuracy of clinical history in patients presenting with reactions to BL (Chiriac et al., 2018), comparison of DHR prevalence in children and adults (Demoly et al., 2012), and NSAIDs patterns of reactions and possible classifications (Caimmi et al., 2012). The objective of this study, based on a national drug allergy registry, was to determine the prevalence, clinical presentation, and drug distribution of DHRs in a country from the Middle East.

## Methods and Materials

Al-Rashed Allergy Center is a tertiary public center in Kuwait, and it is a referral center for all drug allergy evaluation in the country, covering both public and private health systems. An initial drug allergy evaluation is performed on all patients referred to our clinic for suspected DHRs, and patients presenting with a suggestive history of DHRs from July 2007 to June 2019 were included in this study. The following data were collected: patient demographics (age and gender), drug(s) involved in the clinical reaction, signs and symptoms of DHRs (as reported by the patient and/or obtained from their medical records), time of onset of DHRs after drug(s) exposure, results of DPT when indicated, and results of the final evaluation. All patients were evaluated by a detailed clinical history related to ADR or DHR including physical examination (Demoly et al., 1999).

Regarding symptoms, different clinical categories were established; anaphylaxis was defined as per the WHO criteria as a serious allergic reaction that is rapid in onset and might cause death (Simons et al., 2011) and anaphylactic shock, defined as those with anaphylaxis and signs of critical organ hypoperfusion (Thong et al., 2006). Urticaria was defined as hives, angioedema as swelling of the skin, erythema as redness of the skin or mucous membranes, and respiratory symptoms as shortness of breath from upper or lower airways.

Patients were included in the group “multiple” when they refer to the same symptoms upon exposure to three or more different groups of medication. In an attempt to include all groups of drugs that were reported by the patients in our registry, patients were included in the group “others” when they were the only patient in our registry reporting a reaction to a specific group of drugs, and on the contrary, on those cases where more than a single patient refers to symptoms of a specific group of drugs, the group was named by the name of the drug itself (i.e., NSAIDs).

All patients were asked to determine the approximate time elapsed since the intake of the drug and the start of the reaction. Immediate reaction was defined when presenting symptoms, compatible with hypersensitivity reaction, appear till 1 h after drug administration and nonimmediate reaction was defined when presenting symptoms appear after 1 h.

In our cohort, the cultural background of Middle Eastern patients had a strong influence on the way we based our allergy testing. Our patients are usually less prone to assume DPT risks, and they are favoring a safe testing with alternatives, whenever possible.

Patients were grouped according to the following three categories:

1. Confirmed drug allergy: When patients had a positive clinical history alone, defined as symptoms compatible with type I hypersensitivity reactions (immediate), including pruritus, urticaria/angioedema, shortness of breath, on two or more occasions to the same or cross-reacting drugs (graph1), or positive ST/DPT, or if they had an indication for desensitization (Decker et al., 2010).
2. Probable drug allergy: When patients had a single reaction to the offending drug, or in those presenting with severe cutaneous reactions such as SJS/TEN (Stevens–Johnson syndrome/toxic epidermal necrolysis), AGEP (acute generalized exanthematous pustulosis), and DRESS/DIHS/HSS (drug reaction with eosinophilia and systemic symptoms/drug-induced hypersensitivity syndrome/hypersensitivity syndrome).
3. Not confirmed/nonallergic: Patients are defined as not confirmed when they did not consent for DPT, despite being indicated and in case of contraindication for DPT due to comorbidities or other factors: acute infections; cardiac, hepatic, or renal diseases; pregnancy; breastfeeding; or receiving beta-blockers. Patients are defined as nonallergic when they had negative DPT.

**FIGURE 1:**
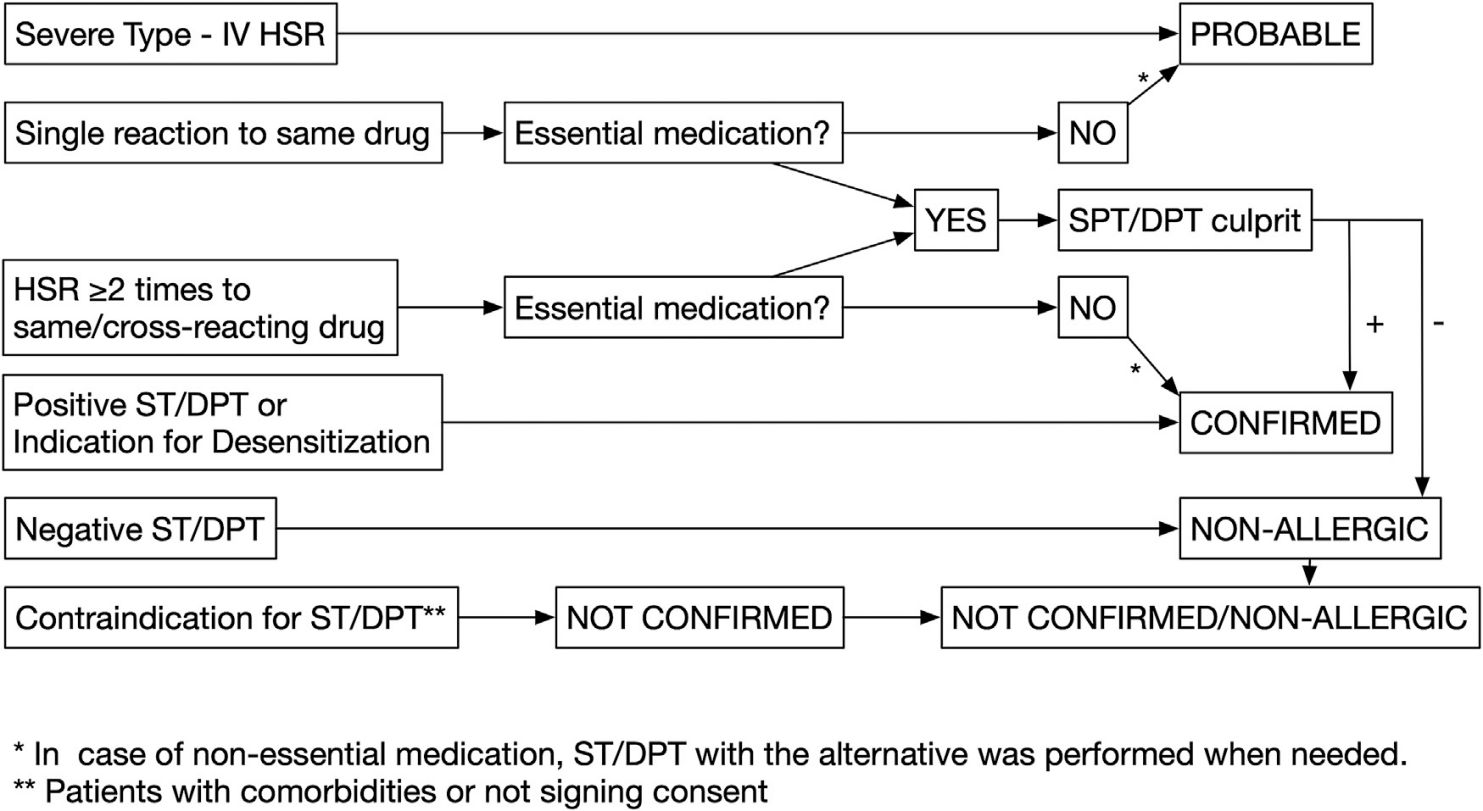
Diagnostic methods flowchart.

An allergy workup was performed on patients with the following drug categories (Figure 1):

1. Essential medications: Antibiotic, NSAIDs, monoclonal antibodies, chemotherapy, proton pump inhibitor, corticosteroids, antidiabetic drugs, antihypertensive drugs, anticoagulants, general anesthesia, anticonvulsants, allopurinol, supplemental drugs (iron and vitamin D), and interferon. Skin tests (STs) and DPT with the culprit drug were used to confirm the diagnosis in these cases. However, when a suitable same efficacy alternative drug is available on those cases with a positive DPT with the culprit drug, additional tests, STs, and DPTs with the suitable alternative were considered in case of cross-reactivity.
2. Nonessential medications: Other supplemental drugs, hyoscine, antihistamines, and local anesthesia. DPT with the culprit drug was not performed, and instead patients were tested with STs and DPTs to a suitable alternative in case of possible cross-reactivity.

On those patients reporting reactions to radio-contrast media (RCM), the diagnosis was confirmed by STs. If STs were negative, a premedication prior to next infusion was recommended (Pichler, 2010 and American Academy of Allergy; Demoly et al., 2014) and desensitization was performed, if premedication fails (Al-Ahmad and Bouza, 2017).

Patients presenting with erythema alone were tested to the offending medication. A risk assessment was performed by the staff for each individual patient presenting with DHRs, and the decision to proceed with STs/DPTs was decided on an individual basis.

## Skin Testing

STs were performed according to the European Network of Drug Allergy (ENDA) (Brockow et al., 2002) guidelines. STs were performed using the dilutions shown in Table 1. The prick test was initially performed, and in those with negative results, was followed by intradermal testing. Intradermal testing is done by marking the bleb created by the injection of 0.3 ml. An immediate positive response was considered when an increase in the diameter of the wheal area was greater than 3 mm than the saline control and accompanied by erythema that is read 15–20 min after testing (Demoly and Bousquet, 2002). A reading was done after 24–48 h in the case of nonimmediate reactions. Patch testing was performed in suspicion of type IV reactions.

**TABLE 1:** Concentrations of the different drugs used for skin prick testing (SPT) and intradermal testing (ID).

Drug	SPT	ID	DPT
PPL	0.04 mg/ml	0.0004–0.04 mg/ml	Amoxicillin/clavulanate 875/125 mg
MDM	0.5 mg/ml	0.005–0.5 mg/ml	Same as above
Amoxicillin	20 mg/ml	0.2 mg/ml	Same as above
Clavulanic acid	5 m-20 mg/ml	0.05–20 mg/ml	Same as above
Ampicillin	25 mg/ml	0.025–25 mg/ml	Same as above
Penicillin G	10,000 U/ml	10–1,000 U/ml	Same as above
Meropenem	1 mg/ml	0.1–1 mg/ml	1 gm
Cephalosporins	2 mg/ml	0.002, 2 mg/ml	Ceftriaxone 2 gm I.V or
			Cefuroxime 500 mg oral
Hydrocortisone	2, 20 mg/ml	0.2, 2 mg/ml	Dexamethasone 5 mg
Methylprednisolone	2, 20 mg/ml	0.2, 2 mg/ml	
Iohexol	350 mg I/ml	35, 350 mg I/mL	NA
Iodixanol	320 mg/ml	3.2, 320 mg/ml	
Lidocaine	20 mg/ml	2 mg/ml	Lidocaine 2 ml 0f 20 mg/ml

**Abbreviations:** PPL, penicilloyl-polylysine; MDM, minor determinant mixture; amoxicillin/clavulanic acid (diater Madrid, Spain), iohexol (GE Health care), ampicillin sodium equivalent to 500 mg ampicillin activity (Bristol-Myers Squibb, United States), Hymox Forte in powder form (Biocheme Spimaco, Saudi Arabia), or amoxicillin commercial kit or clavulanic acid commercial kit (cephalosporin), Penicillin G (Sandoz Gmb H, Kundl-Astria/ Autriche Sanduz), meropenem (AstraZeneca, UK), cefuroxime (Glaxo, Italy), or ceftriaxone (Sandoz, Austria).

## DPT

If STs were negative or not available, DPT with the suspected drug was performed (Aberer et al., 2003; Torres et al., 2003). Single-blind placebo-controlled DPT was performed following the ENDA general guidelines (Torres et al., 2003), with slight modifications in some cases. Drugs were administered at increasing doses every 30–90 min until the full therapeutic dose was reached. In patients with reactions induced by NSAIDs, DPT was performed as previously described (Doña et al., 2011). When patient-reported symptoms (e.g., skin and respiratory) or changes in vital signs were observed (heart rate and blood pressure) or a decrease in the peak expiratory flow rate (PEFR), the procedure was stopped, and patients’ symptoms were evaluated and treated. If patients tolerated the given drug, they were advised to report any nonimmediate reactions and were considered negative DPT.

We followed the general ENDA recommendations for DPT indications, contraindications, prohibited co-medication, and enhanced safety measures (e.g., intravenous catheter) in case of clinical history of anaphylaxis. Uniformed capsules/preparations, including placebo, delivered in specified doses prepared by the hospital pharmacy or commercially available drugs, were used for DPTs. The oral route was chosen systematically, except for drugs with only intravenous or subcutaneous preparations. All DPTs were performed during one day.

## Ethics Committee

All patients were informed about the risk and outcomes of the procedure and provided informed consent. Ethical clearance was granted by Kuwait Research Ethics Committee at the Ministry of Health (Research study number 808/2018).

## Statistical Analysis

Nonparametric and parametric methods are used to calculate statistical significance. The distribution value is determined by D’Agostino and Pearsonomnibus test normality. Student’s t-test, Mann–Whitney test, Fisher’s test, and χ2 test were used for calculating the difference between the groups. The ANOVA test was used to calculate the relative difference distribution variance between variables. The statistical hypotheses were tested at a level of *α* = 0.05, and the difference between the groups in the sample was considered significant when *p* < 0.05 or less. Statistical significance was depicted as *p* < 0.05, *p* < 0.01, and *p* < 0.001. All data were analyzed using GraphPad Prism version 7 (San Diego, California, United States). All the percentages in the tables were calculated from the pooled group of patients, and the difference between pooled groups was calculated using the χ2 test.

## Results

### Description of the Total Sample

We have evaluated 1,553 patients with a history compatible of DHRs, with 65.58% females with a mean age of 41.52 ± 16.93 years. Among all episodes, 42.18% were attributed to antibiotics (32.13% to BLs) and 28.65% to anti-inflammatory drugs and analgesics (24.66% to NSAIDs). Drug allergy was confirmed in 645 (41.5%), probable in 199 (12.8%), and not confirmed/nonallergic in 709 (45.6%) of patients. Of the total studied patients for each drug, confirmation was obtained in 38.10% of the patients for antibiotics (28.20% for BLs) and 39.2% for anti-inflammatory drugs and analgesics (34.10% for NSAIDs) (Table 2).

**TABLE 2:** Clinical characteristics among patients with confirmed and not confirmed/nonallergic.

Clinical characteristic		Confirmed (n = 645; 41.5%)		Probable (n = 199; 12.8%)		Not confirmed/nonallergic (n = 709; 45.6%)		*p* value
Age (years)		42.6 ± 16.02		40.7 ± 17.3		43.1 ± 17.6		0.0625
Females (n; %)		432	66.98	126	63.3	463	65.03	0.6014
Culprit drug	Allopurinol	2	0.3	4	2.0	2	0.3	0.0069^a^
	Anti-inflammatory and analgesics	253	39.2	30	15.1	162	22.8	<0.0001^a^
	Anesthetic	12	1.9	1	0.5	47	6.6	<0.0001^a^
	Antibiotics	246	38.1	72	36.2	337	47.5	0.0004^a^
	Anticholinergic	1	0.2	0	0.0	1	0.1	0.8609
	Anticoagulants	4	0.6	2	1.0	4	0.6	0.7859
	Anticonvulsants	3	0.5	7	3.5	0	0.0	<0.0001^a^
	Antidiabetics	6	0.9	0	0.0	4	0.6	0.3351
	Antihistamines	4	0.6	3	1.5	5	0.7	0.4406
	Antihypertensive	4	0.6	0	0.0	2	0.3	0.3890
	Chemotherapy	4	0.6	3	1.5	2	0.3	0.1299
	Corticosteroids	8	1.2	2	1.0	14	2.0	0.4415
	Hormones	6	0.9	2	1.0	2	0.3	0.2615
	Hyoscine	4	0.6	0	0.0	0	0.0	0.0594
	Interferon	1	0.2	1	0.5	0	0.0	0.2112
	Monoclonal antibodies	7	1.1	3	1.5	3	0.4	0.2209
	Multiple^b^	30	4.7	33	16.6	38	5.4	<0.0001^a^
	Others^c^	32	5.0	25	12.6	16	2.3	<0.0001^a^
	Proton pump inhibitors	8	1.2	2	1.0	2	0.3	0.1221
	Prostaglandin inhibitors	0	0.0	2	1.0	0	0.0	0.0011^a^
	Radio-contrast media	2	0.3	1	0.5	60	8.5	<0.0001^a^
	Supplementals	8	1.2	6	3.0	8	1.1	0.1221
	β lactams^##^	182	28.2	21	10.6	296	41.7	<0.0001^a^
	Quinolones	23	3.6	16	8.0	19	2.7	0.0019^a^
	Macrolides	18	2.8	12	6.0	15	2.1	0.0142^a^
	Sulphomides	13	2.0	7	3.5	3	0.4	0.0021^a^
	Metronidazole	3	0.5	2	1.0	1	0.1	0.2027
	NSAIDs	220	34.1	22	11.1	141	19.9	<0.0001^a^
	Paracetamol	28	4.3	7	3.5	20	2.8	0.3190
	Opioids	5	0.8	1	0.5	1	0.1	0.0282^a^
Clinical symptoms	Urticaria	373	57.8	119	59.8	283	39.9	<0.0001^a^
	Angioedema	274	42.5	132	66.3	226	31.9	<0.0001^a^
	Erythema	217	33.6	173	86.9	285	40.2	<0.0001^a^
	Respiratory	307	47.6	109	54.8	142	20.0	<0.0001^a^
	Anaphylaxis/anaphylactic shock	284	44.0	99	49.7	77	10.9	<0.0001^a^
Timing	Immediate	425	65.9	25	12.6	304	42.9	<0.0001^a^
	Nonimmediate	220	34.1	174	87.4	405	57.1	
Time elapsed between reaction and study	≤1 year	435	67.4	162	81.4	423	59.7	<0.0001^a^
	>1 year	210	32.6	37	18.6	286	40.3	

a Difference was significant statistically.

b *Multiple*: When there was a patient in our registry reporting the same symptoms upon exposure to three or more different groups of medication.

c *Others:* When there was only one patient in our registry reporting a reaction to a specific group of drugs.

### Age and Gender

Patients with confirmed, probable, and not confirmed/nonallergic, with immediate and nonimmediate reactions, and with time elapsed between the reaction and study ≤1 and >1 year showed a similar age and gender distribution (*p* > 0.05) (Table 3).

**TABLE 3:** Clinical characteristics among *all patients* in regard to immediate and nonimmediate drug allergy reactions.

Clinical characteristic		Immediate (n = 754; 48.55%)		Nonimmediate (n = 799; 51.45%)		*p* value
Age(years)		41.3 ± 16.4		40.7 ± 17.4		0.1402
Females (n; %)		497	65.9	524	65.6	0.9148
Drug involved	Antibiotics	308	40.8	347	43.4	0.3045
	Analgesics	267	35.4	178	22.3	<0.0001^a^
	β lactams	251	33.3	248	31.0	0.3556
	NSAIDs	225	29.8	158	19.8	<0.0001^a^
Clinical symptoms	Urticaria	388	51.5	387	48.4	0.2431
	Angioedema	304	40.3	328	41.1	0.7962
	Erythema	237	31.4	438	54.8	<0.0001^a^
	Respiratory	359	47.6	199	24.9	<0.0001^a^
	Anaphylaxis/anaphylactic shock	308	40.8	152	19.0	<0.0001^a^
Time elapsed between reaction and study	≤1 year	506	67.1	514	64.3	0.2616
	>1 year	248	32.9	285	35.7	

a Difference was significant statistically.

### Comparison of Immediate vs. Nonimmediate Reactions

Patients with confirmed allergy showed more frequently immediate reaction (65.90%) than probable drug allergy (12.6%) and not confirmed/nonallergic (42.9%) *p* < 0.0001 (Table 2). Timing of reactions was immediate, ≤1 h, in 48.55% of the patients, and nonimmediate, >1 h, in 51.44%. The ratio of frequency of immediate and nonimmediate reaction was 0.94. In patients with confirmed drug allergy, the frequency ratio of immediate and nonimmediate reactions was 1.93 (Table 4). In patients with confirmed drug allergy, allergy to antibiotics and BLs was more common in patients with immediate reaction, while in patients with nonimmediate reaction, hypersensitivity to analgesics and NSAIDs was more common (Table 4). Among patients with confirmed drug allergy, immediate and nonimmediate reactions were similarly distributed between allergy to antibiotics and analgesics (Table 5).

**TABLE 4:** Clinical characteristics among patients with confirmed drug allergy in regard to immediate and nonimmediate drug allergy reactions.

Clinical characteristic		Immediate (n = 425; 65.9%)		Non-immediate (n = 220; 34.1%)		*p* value
Age (years)		42.7 ± 15.7		41.1 ± 16.6		0.8995
Females (n; %)		293	68.9	139	63.2	0.1577
Drug involved	Antibiotics	171	40.2	59	26.8	0.0007^a^
	Analgesics	194	45.6	75	34.1	0.0054^a^
	β lactams	134	31.5	48	21.8	0.0098^a^
	NSAIDs	167	39.3	53	24.1	0.0001^a^
Clinical symptoms	Urticaria	249	58.6	124	56.4	0.6142
	Angioedema	194	45.6	80	36.4	0.0288^a^
	Erythema	126	29.6	91	41.4	0.0037^a^
	Respiratory	267	62.8	40	18.2	<0.0001^a^
	Anaphylaxis/anaphylactic shock	248	58.4	36	16.4	<0.0001^a^
Time elapsed between reaction and study	≤1 year	286	67.3	149	67.7	0.9296
	>1 year	139	32.7	71	32.3	

a Difference was significant statistically.

**TABLE 5:** Frequency of symptoms and reactions on specific drug allergy is done in confirmed allergy only.

		Antibiotics (n = 246)		Analgesics (n = 253)		*p* value	β lactams (n = 182)		NSAIDs (n = 220)		*p* value
		n	%	N	%		n	%	n	%	
Symptoms	Urticaria	161	65.4	130	51.4	0.0015^a^	119	65.4	108	49.1	0.0012^a^
	Angioedema	103	41.9	117	46.2	0.3672	75	41.2	102	46.4	0.3142
	Erythema	84	34.1	75	29.6	0.2918	56	30.8	61	27.7	0.5103
	Respiratory symptoms	123	50.0	140	55.3	0.2446	101	55.5	124	56.4	0.9197
	Anaphylaxis/anaphylactic shock	125	50.8	107	42.3	0.0599	103	56.6	91	41.4	0.0026^a^
Timing	Immediate reaction	171	69.5	194	76.7	0.0857	134	73.6	167	75.9	0.6445
	Nonimmediate reaction	75	30.5	59	23.3		48	26.4	53	24.1	

a Difference was significant statistically.

Note: These symptoms do overlap.

### Time Since Reaction to Study

Patients with confirmed drug allergy showed more frequent time elapsed between the reaction and study ≤1 year than not confirmed/nonallergic patients, but less frequent than patients with probable allergy (Table 2). The time elapsed between reaction and study >1 year was similar to the ones without anaphylaxis/anaphylactic shock (Table 6).

**TABLE 6:** Clinical characteristics of patients with confirmed drug allergy in regard to anaphylaxis.

Clinical characteristic		With anaphylaxis/anaphylactic shock (n = 284; 44.03%)		Without anaphylaxis/anaphylactic shock (n = 361; 55.97%)		*p* value
Age (years)		43.4 ± 15.7		46.2 ± 16.3		0.6409
Females (n; %)		206	72.5	226	62.6	0.0089^a^
Drug involved	Antibiotics	125	44.0	121	33.5	0.0071^a^
	Analgesics	107	37.7	146	40.4	<0.0001^a^
	β lactams	103	36.3	79	21.9	0.3576
	NSAIDs	91	32.0	129	35.7	<0.0001^a^
Timing	Immediate	248	87.3	177	49.0	<0.0001^a^
	Nonimmediate	36	12.7	184	51.0	
	≤1 year	198	69.7	237	65.7	0.3098
	>1 year	86	30.3	124	34.3	

a Difference was significant statistically.

### Comparison of Confirmed and Not Confirmed Cases

Patients with confirmed drug allergy showed more frequent (*p* < 0.05) allergy to analgesics, NSAIDs, and opioids than patients with probable drug allergy and not confirmed/nonallergic. However, patients with probable drug allergy showed more frequent allergy to allopurinol, anticonvulsants, multiple drugs, other drugs, prostaglandin inhibitors, quinolones, macrolides, and sulphomides than patients with confirmed and not confirmed/nonallergic. Furthermore, not confirmed/nonallergic patients showed more frequent allergy to anesthetics, antibiotics, radio-contrast media, and β lactams than patients with probable and confirmed drug allergy (Table 2).

All symptoms (urticaria, angioedema, respiratory symptoms, and anaphylaxis) were more common in patients with confirmed and probable drug allergy, rather than in not confirmed/nonallergic patients, with the exception of erythema, which was most common in patients with probable allergy (Table 2). Anaphylaxis was shown in 44.00% of confirmed patients, which was 18.28% of the total population (Table 2). In this group of patients, angioedema, erythema, respiratory symptoms, and anaphylaxis were similarly distributed in allergy to antibiotics and analgesics. However, urticaria was more frequent in allergy to antibiotics than in analgesics allergy. Urticaria and anaphylaxis were more common in BL than NSAID hypersensitivity, while angioedema, erythema, respiratory symptoms, and immediate and nonimmediate reactions were similarly distributed between BL and NSAID hypersensitivity (Table 5). In patients with confirmed drug allergy, patients with anaphylaxis were younger than those without anaphylaxis, but these differences were not statistically significative (*p* = 0.6409) (Table 6). Patients with anaphylaxis/anaphylactic shock showed more common allergy to antibiotics, but less common to analgesics and NSAIDs, than patients without anaphylaxis/anaphylactic shock (Table 6). Among the anaphylactic cases, antibiotics were the culprit in 44% of cases, whereas anti-inflammatory drugs and analgesics as a group was responsible in 37.7% (<0.0001) (Table 6).

In regard to diagnosis, the most common method was a positive clinical history (54.4% in BLs and 90.45% in nonsteroidal anti-inflammatory drugs (NSAIDs)). Among confirmed allergy to BLs, positive ST was obtained in 31.9% of patients and positive DPT in 13.7% (Table 7). Among patients with confirmed drug allergy, allergy diagnosis was made more frequently by positive history alone for the following drugs: BLs, quinolones, macrolides, metronidazole, sulphomides, NSAIDs, paracetamol, opioids, and RCM (Table 7).

**TABLE 7:** Diagnostic methods in confirmed drug allergy.

Drug	Positive by history only		Positive by skin prick test		Positive by DPT		*p* value
	n	%	n	%	n	%	
β lactams (n = 182)	99	54.4	58	31.9	25	13.7	<0.0001^a^
Quinolones (n = 23)	20	86.9	0	0.0	3	13.1	<0.0001^a^
Macrolides (n = 18)	18	100.0	0	0.0	0	0.0	<0.0001^a^
Metronidazole (n = 3)	3	100.0	0	0.0	0	0.0	<0.0001^a^
Sulphomides (n = 13)	13	100	0	0.0	0	0.0	<0.0001^a^
NSAIDs (n = 220)	199	90.45	0	0.0	9	9.54	<0.0001^a^
Paracetamol (n = 28)	25	89.29	0	0.0	3	10.71	<0.0001^a^
Opioids (n = 5)	5	100	0	0.0	0	0.0	<0.0001^a^
RCM (n = 2)^b^	0	0.0	0	0.0	0	0.0	—

a Difference was significant statistically.

b Desensitization was done for 12 patients who had reaction to NSAIDs.

c Desensitization was done for two patients who had reaction to RCM.

DPT, drug provocation test.

## Discussion

To our knowledge, this is the first published drug allergy database in the Middle East region. We hypothesize that this might be due not only to the complexity and time-consuming task of developing a standardized database in a registry-based format (Bousquet et al., 2009) but also to other factors as the recently developed electronic databases, the relatively recent increased development of the health systems in Middle East compared with those in Europe and North America. This is a drug allergy registry–based study that was done over 12-year duration. The diagnosis in our study was confirmed in 41.5% of cases, and this compares to other European studies where drug allergy was confirmed in 37.4% and not confirmed/nonallergic in 62.6% (including 13.4% with contraindications for testing) (Doña et al., 2012), and American studies, where at least one drug allergy was confirmed in 19.66% of patients (Blanca et al., 2020). This difference can be attributed to the confirmation criteria that were adapted in our study, which specifically include patients who had a positive clinical history alone. This is a key factor to understand some of the diagnostic differences with other studies.

Our sex and age distribution is similar to other studies that report 64.58–71.9% of females, with a mean age of 43.7–48.9 years (Doña et al., 2012; Gabrielli et al., 2018; Blanca et al., 2020). In our cohort, reactions occurred ≤1 h in 48.55% of all the patients and ≥1 h in 51.44%. Interestingly, these results are similar to those of a study by Bousquet PJ et al. (Bousquet et al., 2008), which excluded type IV reactions and found that reactions occurring ≤1 h after drug intake in 36.6% of patients. However, other studies (Ben-Shoshan et al., 2018) focused on BL reactions and found a reaction ≤1 h after drug intake in 19.9% and after 24 h in 34.4%.

Many studies have evaluated the timing since reaction; interestingly, the average delay was 299.7 months in a large study, which was reduced to 43 months on those confirmed for one drug and 76.9 months for multiple drugs (Blanca et al., 2020). On the other hand, a study with BLs showed an average of 54.7 months for not confirmed/nonallergic and 25.8 months for confirmed patients (Bousquet et al., 2008). These results are consistent with ours, with confirmed and probable patients presenting earlier to our clinic for consultation.

The number of confirmed and probable patients showing a time elapse since reaction <1 year was significantly higher than for those not confirmed/nonallergic, and this is consistent with other studies following the decrease in positive ST responses after 1, 3, and 5 years which showed a decrease of 68.1, 50, and 36.1% for cephalosporins (Demoly et al., 2003) or 80.6, 78.3, and 70.6% for patients presenting positive STs to benzylpenicilloyl (BPO) or minor determinant mixture (MDM) or 50, 54, and 0% for patients reacting to amoxicillin side chains (Blanca et al., 1999). This decrease in sensitivity over time has also been reported in NSAIDs for NIUA (NSAID-induced urticaria/angioedema) and SNIUAA (single NSAID-induced urticaria/angioedema and anaphylaxis) (Doña et al., 2020).

Of the total studied patients for each drug, confirmation was obtained in 42.17% of the patients with antibiotics (32.13% for BLs) and 28.65% with anti-inflammatory drugs and analgesics (24.66% for NSAIDs). In other studies, hypersensitivity to NSAIDs was confirmed in 19.6–27% and BLs in 18.4–6.99% (Bourke et al., 2015; Cornejo-García et al., 2019; Blanca et al., 2020). Confirmation was reached for BLs in 45.6% of the patients by means of STs or DPTs, and the remaining by clinical history alone. When compared with American studies of patients allergic to BLs, one study showed that 7.35% of tested individuals had positive penicillin ST results, with only 1.6% of the negative ST patients had a reaction to the DPT (Macy et al., 2009). Another study (Blanca et al., 2020) showed that 14.14% of tested individuals had positive ST or DPT results. In an Australian study that evaluated the effectiveness of penicillin allergy delabeling of 341 patients, a positive ST was found in 42 (12.3%) of patients, which was similar to our findings, in which 58 of 499 BL patients (11.62%) had positive STs (Bourke et al., 2015). In comparison to European studies, our results compare with a multicenter study that included patients with reactions to BLs only (Chiriac et al., 2018; Ben-Shoshan et al., 2018), in which 23.6% of the studied patients were confirmed as allergic by means of STs or DPTs only. Furthermore, the number of confirmed patients in these studies was lower than ours as positive testing was generally required for confirmation. Previous studies from our group showed that our data compare more with European than with American studies (Al-Ahmad et al., 2014; Al-Ahmad and Rodriguez-Bouza, 2018); this multicenter study can provide an idea of additional patient’s number required to be positive using our criteria of positive BL allergy based on clinical history alone.

The frequency of drug allergy types varied among different studies. In some studies (Doña et al., 2012; Çelik et al., 2014), 31.9–37% of the episodes were attributed to NSAIDs and 20.4–28.1% to BL antibiotics (Doña et al., 2012; Çelik et al., 2014; Gabrielli et al., 2018), and the most frequent drug allergy was to multiple NSAIDs 47.29%, followed by immediate reactions to BLs 18.12% (Doña et al., 2012), and these findings are similar to ours. However, another study (Gabrielli et al., 2018) reported 20.3% of patients were confirmed to NSAIDs, which was lower than our study, and 57.8% of the reactions were due to antibiotics, which was higher than ours.

In the study by Doña et al. (Doña et al., 2012), the diagnosis was established by clinical history in 742 patients (44%), by SPTs in 246 patients (14.6%), by *in vitro* testing in 176 patients (10.4%), and by DPT in 519 patients (30.8%). This was different from our results. We had about 79.58% patients diagnosed by clinical history alone, 12.08% by SPT, and 8.33 by DPT. These differences are explained by the escalating preferences of patients for more conservative approaches including alternative treatments, rather than performing DPT with the culprit drug.

STs or serum-specific IgE antibodies were used as methods of diagnosis in BL reactions in 70–82% of patients and DPT in 18–30% (Bousquet et al., 2008; Kalyoncu et al., 2016), and this was in concordance with our findings, where the method to confirm diagnosis in BLs was STs in 31% of patients and a DPT in 13.7%. The observed difference was likely due to not performing STs in patients with anaphylactic reactions to BLs, and instead performing drug testing with suitable alternatives (Al-Ahmad et al., 2014; Al-Ahmad and Rodriguez-Bouza, 2018). Of the five confirmed patients for opioids reactions, the diagnosis was elucidated from a positive history only (100% of patients), compared to other studies, where they used DPT as the main diagnostic method. These differences are due to a more conservative approach where a suitable alternative could be found (Li et al., 2017; Powell et al., 2019).

We are aware of some limitations in our study. The most important limitation of the study is the mixing of unconfirmed and nonallergic patients in the same category. Our group was forced to do so because we were using real-life data from a registry, and even if the outcome of the test is very likely to be negative, patients who rejected or had contraindication for DPT cannot be called nonallergic, and patients with negative DPT are simply “nonallergic” and cannot be called not confirmed. The use of clinical history alone as a positive criterion should not be used if we rely on other diagnostic testing. Another limitations of the study included that some patients were unsure on which drug caused the reaction, the temporal correlation after drug exposure due to recall bias, the relatively small study population, especially for less common drug reactions, and that atopy was not routinely assessed in all patients, and therefore, atopy could not be studied as a risk factor. However, this is a prospective data-based registry, and ongoing data collection might address some of these issues in the near future.

## Data Availability Statement

The raw data supporting the conclusions of this article will be made available by the authors, without undue reservation.

## Ethics Statement

The studies involving human participants were reviewed and approved by Kuwait Research Ethics Committee at the Ministry of Health (Research study number 808/2018). Written informed consent from the participants' legal guardian/next of kin was not required to participate in this study in accordance with the national legislation and the institutional requirements.

## Author Contributions

All authors listed have made a substantial, direct, and intellectual contribution to the work and approved it for publication.

## Conflict of Interest

The authors declare that the research was conducted in the absence of any commercial or financial relationships that could be construed as a potential conflict of interest.
